# Association between visceral adiposity index and sleep disorders among the U.S. adults: a cross-sectional study

**DOI:** 10.3389/fneur.2025.1540182

**Published:** 2025-05-09

**Authors:** Chunhua Liu, Linan Qiu, Tingting Wang, Zegen Ye, Simin Wu, Di Li, Huajian Lin, Yue Jin

**Affiliations:** ^1^Lishui Hospital of Traditional Chinese Medicine Affiliated to Zhejiang University of Chinese Medicine, Liandu District, Lishui, Zhejiang, China; ^2^Department of Geriatrics and Neurology, Affiliated Hospital and Yuying Children's Hospital, Wenzhou Medical University, Wenzhou, China

**Keywords:** sleep disorders, visceral adiposity index, cross-sectional study, NHANES, sleep

## Abstract

**Background:**

The visceral adiposity index (VAI) reliably measures body fat distribution and related dysfunctions. However, its association with sleep disorders among US adults remains unclear.

**Methods:**

This study analyzed cross-sectional data from the 2005 to 2018 National Health and Nutrition Examination Survey (NHANES) for adults aged 18 and older. We used multivariable logistic regression to evaluate the association between VAI and sleep disorders and applied restricted cubic splines to assess potential non-linear relationships. Additionally, subgroup analyses by gender, age, and race were conducted to explore the VAI-sleep disorder association across different populations.

**Results:**

This study included 14,021 adults aged 18 +. In Model 1, adjusted for gender and age, each unit increase in VAI was associated with a 5% higher risk of sleep disorders (OR = 1.05; 95% CI = 1.02–1.07). In Model 2, which adjusted for all potential confounders, each unit increase in VAI was linked to a 3% higher risk (OR = 1.03; 95% CI = 1.00–1.05). When treating VAI as a categorical variable, those in the highest quartile (Q4) had a 21% higher risk of sleep disorders compared to those in the lowest quartile (Q1) (OR = 1.21; 95% CI 1.03–1.41). Restricted cubic spline analysis revealed a positive linear relationship between VAI and sleep disorder prevalence. Subgroup analysis found stronger associations in males and non-Hispanic white individuals.

**Conclusion:**

While causality cannot be confirmed, this cross-sectional study shows a significant positive linear association between higher VAI and the risk of sleep disorders among U.S. adults.

## 1 Introduction

Sleep is a universal function that occupies one-third of human life. An analysis by the Centers for Disease Control and Prevention (CDC) indicates that the significant decrease in adult sleep duration from 1985 to 2012 has become a public health issue (1). A recent study shows that 27.1% of American adults suffer from sleep disorders (2). Insufficient or poor-quality sleep is associated with a range of bodily dysfunctions. Disorders such as insomnia, sleep apnea, and restless legs syndrome disrupt normal sleep patterns and can have a substantial impact on both mental and physical wellbeing (3). These disorders are associated with negative health outcomes, including obesity, hypertension, cardiovascular diseases, and increased mortality (4–6). Due to the high prevalence of sleep disorders and their connection to these adverse health outcomes, identifying the risk factors for sleep disorders is essential.

Obesity is a major risk factor for the development and progression of certain sleep disorders, such as obstructive sleep apnea (OSA), although it is not a risk factor for all sleep disorders. For every 6-unit increase in body mass index (BMI), the risk of OSA increases four-fold (7). OSA is characterized by recurrent airway obstructions that lower blood oxygen levels. Excess weight may hinder normal breathing, contributing to OSA and symptoms such as frequent nighttime awakenings and reduced sleep duration (8, 9). Visceral adipose tissue secretes pro-inflammatory cytokines, including IL-1, IL-6, and TNF-α, which are known to contribute to chronic inflammation and affect sleep regulation (10). TNF-α and IL-1β peak at night and play an important role in slow-wave sleep (11, 12). In overweight and obese individuals, these cytokines are elevated in the morning and have been associated with sleep disorders and increased BMI (13). Thus, obesity may influence sleep by altering inflammatory factors. Furthermore, waist circumference (WC) is a strong predictor of sleep disorders as it reflects visceral fat accumulation, suggesting that visceral fat may further contribute to sleep problems. However, BMI and similar measures cannot differentiate between fat, muscle, and bone, which may lead to the misclassification of excess fat (14, 15).

Emerging research shows that fat distribution is a critical factor in chronic diseases (16, 17). The visceral adiposity index (VAI) combines HDL, triglycerides (TG), BMI, and waist circumference to serve as a robust indicator of visceral fat and its related dysfunction (18, 19). Although accurate, methods such as Computed Tomography (CT) and Magnetic Resonance Imaging (MRI) for detecting visceral obesity are costly and time-consuming, and CT involves radiation, making these techniques impractical for large-scale screening (20). VAI, which incorporates both body measurements and metabolic parameters, is a practical and reliable measure for assessing fat distribution and function. Research has shown that VAI is strongly linked to cardiovascular events and atherosclerosis, and it is an independent risk factor for coronary artery disease, hypertension, and diabetes (21–23). It offers a superior alternative to traditional obesity measures like BMI and WC in distinguishing between subcutaneous and visceral fat.

This study investigates the relationship between the VAI and the prevalence of sleep disorders in American adults using data from the National Health and Nutrition Examination Survey (NHANES) collected between 2005 and 2018. Our objective is to provide scientific evidence that supports the prevention, diagnosis, and treatment of sleep disorders while validating earlier research findings.

## 2 Materials and methods

### 2.1 Study design and population

**Figure 1:**
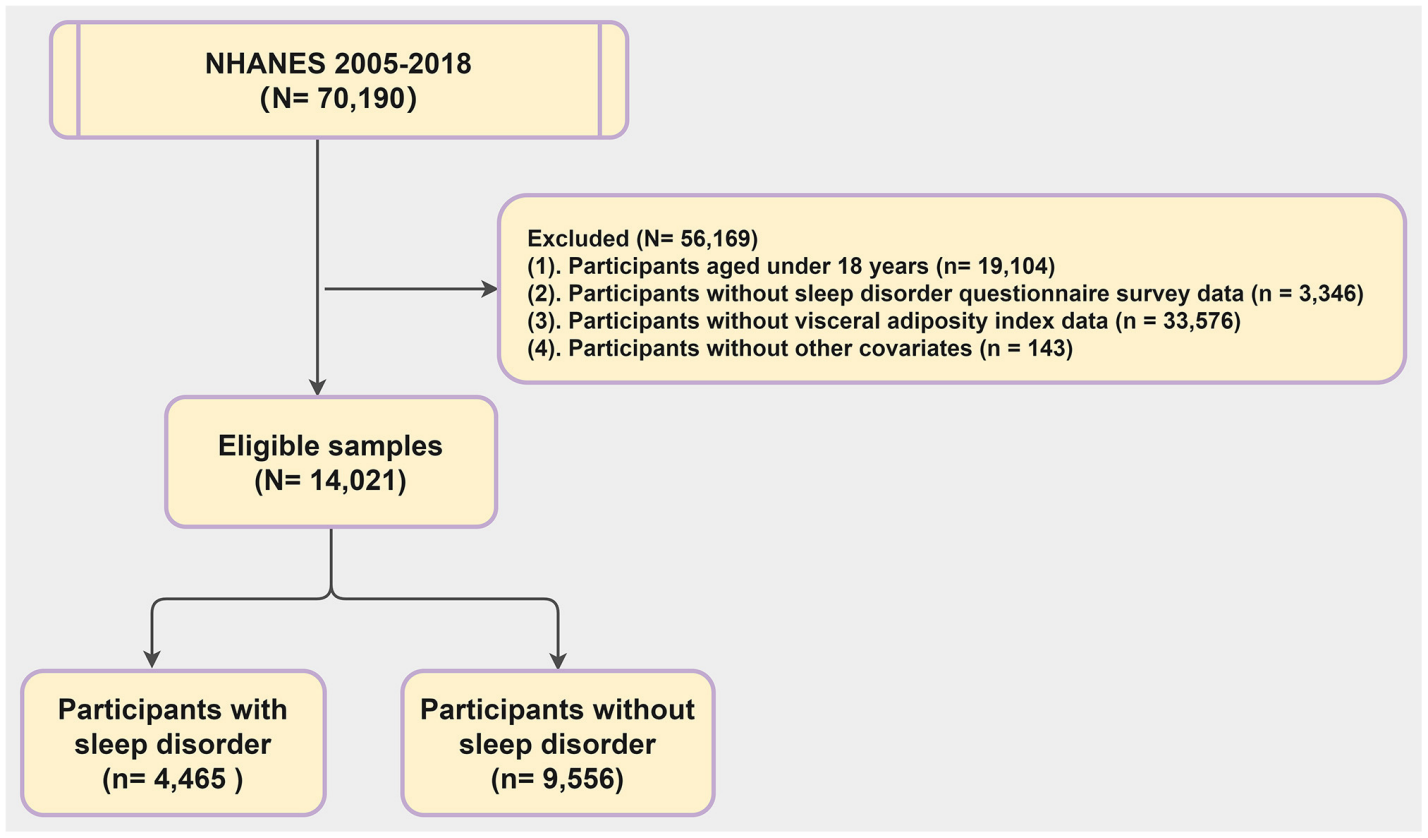
Process for screening participants.

The NHANES is a large cross-sectional survey assessing the health and nutritional status of American adults and children. Data for our study were obtained from the NHANES website (https://www.cdc.gov/nchs/nhanes/index.htm) with informed consent from participants and approval from the National Center for Health Statistics (NCHS) Ethics Review Board. We focused on data from the seven most recent NHANES cycles (2005–2018). Out of 70,190 participants, 14,021 were included in our analysis. The rest were excluded due to being under 18 (19,104 individuals), missing sleep questionnaire data (3,346 individuals), lacking VAI data (33,576 individuals), or incomplete covariate information (5,143 individuals) (Figure 1).

### 2.2 Definition of VAI

The collection and measurement of blood samples in NHANES follow standardized protocols set by the CDC. A dedicated team manages data organization, specimen testing, and analysis. The VAI evaluates visceral fat using anthropometric measures (BMI and WC) and biochemical markers (TG and HDL-C). VAI is calculated based on gender-specific formulas established by Amato et al. (24), where TG and HDL are measured in mmol/L, WC in cm, and BMI in kg/m>. The formula is as follows:


$$\begin{array}{l} \;Males:VAI=(\frac{WC}{39.68\;+1.88^{*}BMI}{)}^{*}(\frac{TG}{1.03}{)}^{*}(\frac{1.31}{HDL})\\ Females:VAI=(\frac{WC}{36.58\;+1.89^{*}BMI}{)}^{*}(\frac{TG}{0.81}{)}^{*}(\frac{1.52}{HDL}) \end{array}$$

### 2.3 Definition of sleep disorders

To assess sleep disorders, we employed a comprehensive three-dimensional sleep questionnaire based on the rigorous NHANES protocol, as used in previous research (5, 25). In-home interviews regarding sleep disorders were conducted using the Computer Assisted Personal Interviewing (CAPI) system, which includes online help screens and consistency checks to enhance data accuracy. During these interviews, trained interviewers asked participants whether a doctor or health professional had ever diagnosed them with a sleep disorder. Participants who answered “yes” were classified as having a sleep disorder. Although this general inquiry does not distinguish between specific types of sleep disorders, it reflects the standardized NHANES protocol and provides a reliable measure based on professional medical diagnoses.

### 2.4 Covariate definitions

In our study, we included several covariates: age, race/ethnicity, gender, educational level, and poverty-to-income ratio (PIR). Additionally, we considered smoking status, alcohol consumption, hypertension, diabetes, stroke, cardiovascular disease, and dietary caffeine intake.

In this study, race was classified into five groups: Mexican American, non-Hispanic Black, non-Hispanic White, other Hispanic, and other races. The poverty income ratio (PIR) was divided into three groups: <1.30, 1.30her Hispanic, and other races. The poverty income ratio (PIR) was divs. “above high school.” BMI was grouped as underweight (BMI < 18.5 kg/mratio (PIR) was divided 8.5 to < 25 kg/m^2^), overweight (BMI 25 to < 30 kg/m^2^), and obese (BMI ≥ 30 kg/m^2^). Smoking status was defined as current smokers (those who have smoked at least 100 cigarettes and still smoke), former smokers (those who have smoked at least 100 cigarettes but no longer smoke), and never smokers (those who have never smoked or smoked fewer than 100 cigarettes). Alcohol consumption was assessed via a questionnaire, classifying participants as non-drinkers, 1–5 drinks/month, 5–10 drinks/month, or 10+ drinks/month. Diabetes mellitus was diagnosed based on a glycated hemoglobin level above 6.5% or a fasting blood glucose level of 7 mmol/L or higher. Stroke, hypertension, and cardiovascular disease were self-reported by participants. Cardiovascular disease in this study included conditions such as coronary artery disease, heart failure, angina pectoris, and heart attack.

### 2.5 Statistical analysis

We obtained NHANES data from the 2005 to 2006, 2007 to 2008, 2009 to 2010, 2011 to 2012, 2013 to 2014, 2015 to 2016, and 2017 to 2018 cycles for our analysis. We assessed the normality of continuous variables during data processing and found that they were all non-normally distributed. Consequently, we reported these non-normally distributed continuous variables as medians with interquartile ranges (IQR), while categorical variables were presented as unweighted frequencies and weighted percentages.

We used a weighted logistic regression model to investigate the link between VAI and sleep disorders, presenting findings as adjusted odds ratios (ORs) with 95% confidence intervals (CIs). Model 1 adjusted for gender and age, while Model 2 additionally accounted for race, education level, poverty-to-income ratio (PIR), alcohol use, smoking status, hypertension, diabetes, stroke, cardiovascular disease, and dietary caffeine intake. Multicollinearity among covariates was evaluated using the variance inflation factor. We treated VAI quartile medians as continuous variables to assess linear trends and applied a restricted cubic spline (RCS) term with the “rcssci” R package (26), optimizing the number of knots based on the Akaike Information Criterion (AIC). Subgroup analyses were performed by age, sex, and ethnicity to explore the impact of VAI on sleep disorders in different populations. Missing covariate data were addressed through multiple imputation using the R package “mitml” (26), with statistical significance set at *P* < 0.05.

## 3 Results

### 3.1 Baseline characteristics

The key characteristics of the study population are detailed in Table 1. This study included 14,021 participants, with a weighted average age of around 47 years. Among these, 35% reported experiencing sleep disorders, with 42% being male and 58% female. The findings suggest that those at higher risk of sleep disorders tend to be female, aged 40 or older, non-Hispanic white people, more educated, with higher BMI and WC, increased caffeine consumption, and falling into the third and fourth quartiles (Q3 and Q4) of the VAI. Moreover, individuals with sleep disorders were more likely to have hypertension. Comparing participants with and without sleep disorders, significant differences were found between the groups in terms of age, race, education level, smoking habits, alcohol consumption, BMI, WC, and TG levels (*P* < 0.05).

**Table 1:** Characteristics of the study population according to sleep disorders.

**Characteristic**	**N^a^**	**Overall, *N =* 14,021 (100%)**	**Group**		***P*-value^c^**
			**No sleep disorder**, ***N** =* **9,556 (65%)**^b^	**Sleep disorder**, ***N** =* **4,465 (35%)**^b^	
Age (years), Median (Mean, SD)	14,021	47 (47, 17)	44 (45, 17)	51 (51, 16)	< 0.001
**Age group**, ***n*** **(%)**	14,021				< 0.001
18–39 years		5,158 (37%)	4,045 (42%)	1,113 (24%)	
40–59 years		4,482 (32%)	2,806 (30%)	1,676 (38%)	
≥60 years		4,381 (31%)	2,705 (28%)	1,676 (38%)	
**Gender**, ***n*** **(%)**	14,021				< 0.001
Female		7,262 (52%)	4,656 (49%)	2,606 (58%)	
Male		6,759 (48%)	4,900 (51%)	1,859 (42%)	
**Race**, ***n*** **(%)**	14,021				< 0.001
Mexican American		2,279 (16%)	1,782 (19%)	497 (11%)	
Other Hispanic		1,345 (10%)	942 (10%)	403 (10%)	
Non-Hispanic White		6,194 (44%)	3,910 (41%)	2,284 (51%)	
Non-Hispanic Black		2,891 (21%)	1,982 (21%)	909 (20%)	
Other Races		1,312 (9%)	940 (9%)	372 (8%)	
**Education level**, ***n*** **(%)**	14,021				< 0.001
≤ High school		3,405 (24%)	2,386 (25%)	1,019 (23%)	
>High school		9,796 (70%)	6,477 (68%)	3,319 (74%)	
Missing data		806 (6%)	693 (7%)	113 (3%)	
The ratio of family income to poverty, Median (Mean, SD)	14,021	2.95 (2.97, 1.64)	2.98 (2.98, 1.63)	2.91 (2.97, 1.66)	0.8
**The ratio of family income to poverty**, ***n*** **(%)**	14,021				0.59
< 1.30		4,237 (31%)	2,820 (30%)	1,417 (32%)	
1.30–3.5		4,829 (34%)	3,350 (35%)	1,479 (33%)	
>3.5		3,834 (27%)	2,612 (27%)	1,222 (27%)	
Missing data		1,121 (8%)	774 (8%)	347 (8%)	
Body Mass Index (kg/m^b^), Median (Mean, SD)	14,021	28 (29, 7)	27 (28, 6)	29 (30, 7)	< 0.001
**Body Mass Index group (kg/m**^b^**)**, ***n*** **(%)**	14,021				< 0.001
Underweight (< 18.5)		256 (2%)	187 (2%)	69 (2%)	
Normal (18.5 to < 25)		4,060 (29%)	3,034 (32%)	1,026 (22%)	
Overweight (25 to < 30)		4,586 (33%)	3,263 (34%)	1,323 (30%)	
Obese (30 or greater)		5,119 (36%)	3,072 (32%)	2,047 (46%)	
Waist Circumference (cm), Median (Mean, SD)	14,021	97 (99, 17)	96 (97, 16)	101 (102, 18)	< 0.001
Total Cholesterol (mg/dL), Median (Mean, SD)	14,021	190 (194, 42)	190 (193, 41)	191 (194, 43)	0.4
Triglyceride (mg/dL), Median (Mean, SD)	14,021	105 (129, 108)	102 (126, 106)	110 (136, 111)	< 0.001
HDL-Cholesterol (mg/dL), Median (Mean, SD)	14,021	52 (54, 16)	52 (54, 16)	52 (54, 17)	0.69
LDL-cholesterol (mg/dL), Median (Mean, SD)	14,021	111 (114, 35)	111 (114, 35)	110 (113, 36)	0.2
Dietary caffeine intake, Median (Mean, SD)	14,021	120 (168, 181)	111 (158, 173)	140 (187, 194)	< 0.001
**Smoking status**, ***n*** **(%)**	14,021				< 0.001
Current		2,808 (21%)	1,697 (18%)	1,111 (25%)	
Former		3,312 (25%)	2,068 (22%)	1,244 (29%)	
Non-smoker		7,901 (55%)	5,791 (59%)	2,110 (45%)	
**Alcohol consumption status**, ***n*** **(%)**	14,021				0.13
Non-drinker		3,396 (24%)	2,445 (26%)	951 (21%)	
1–5 drinks/month		5,916 (42%)	4,094 (43%)	1,822 (41%)	
5–10 drinks/month		922 (7%)	674 (6%)	248 (6%)	
10+ drinks/month		1,710 (12%)	1,216 (13%)	494 (11%)	
Missing data		2,075 (15%)	1,127 (12%)	948 (21%)	
**Diabetes**, ***n*** **(%)**	14,021				< 0.001
Yes		2,414 (17%)	1,345 (14%)	1,069 (24%)	
No		11,607 (83%)	8,211 (86%)	3,396 (76%)	
**Hypertension group**, ***n*** **(%)**	14,021				< 0.001
Yes		4,943 (35%)	2,750 (29%)	2,193 (49%)	
No		9,060 (65%)	6,791 (71%)	2,269 (51%)	
Missing data		18 (< 0.1%)	15 (< 0.1%)	3 (< 0.1%)	
**Cardiovascular disease**, ***n*** **(%)**	14,021				< 0.001
Yes		580 (4%)	272 (3%)	308 (7%)	
No		12,617 (90%)	8,595 (90%)	4,022 (90%)	
Missing data		824 (6%)	689 (7%)	135 (3%)	
**Stroke group**, ***n*** **(%)**	14,021				< 0.001
Yes		498 (4%)	233 (2%)	265 (6%)	
No		12,702 (90%)	8,639 (90%)	4,063 (91%)	
Missing data		821 (6%)	684 (8%)	135 (3%)	
Visceral adiposity index, Median (Mean, SD)	14,021	1.45 (2.07, 2.69)	1.38 (1.95, 2.45)	1.60 (2.28, 3.07)	< 0.001
**Visceral adiposity index, quartile**, ***n*** **(%)**	14,021				< 0.001
Q1 ( ≤ 0.877)		3,506 (25%)	2,549 (26%)	957 (23%)	
Q2 (0.877–1.441)		3,505 (25%)	2,474 (27%)	1,031 (22%)	
Q3 (1.441–2.445)		3,505 (25%)	2,325 (24%)	1,180 (26%)	
Q4 (≥2.445)		3,505 (25%)	2,208 (23%)	1,297 (29%)	

^*a*^ N not Missing (unweighted).

^*b*^ Median (IQR) for continuous; n (%) for categorical.

^*c*^ Wilcoxon rank-sum test for complex survey samples; chi-squared test with Rao and Scott's second-order correction.

### 3.2 Multifactorial logistic regression analysis

**Figure 2:**
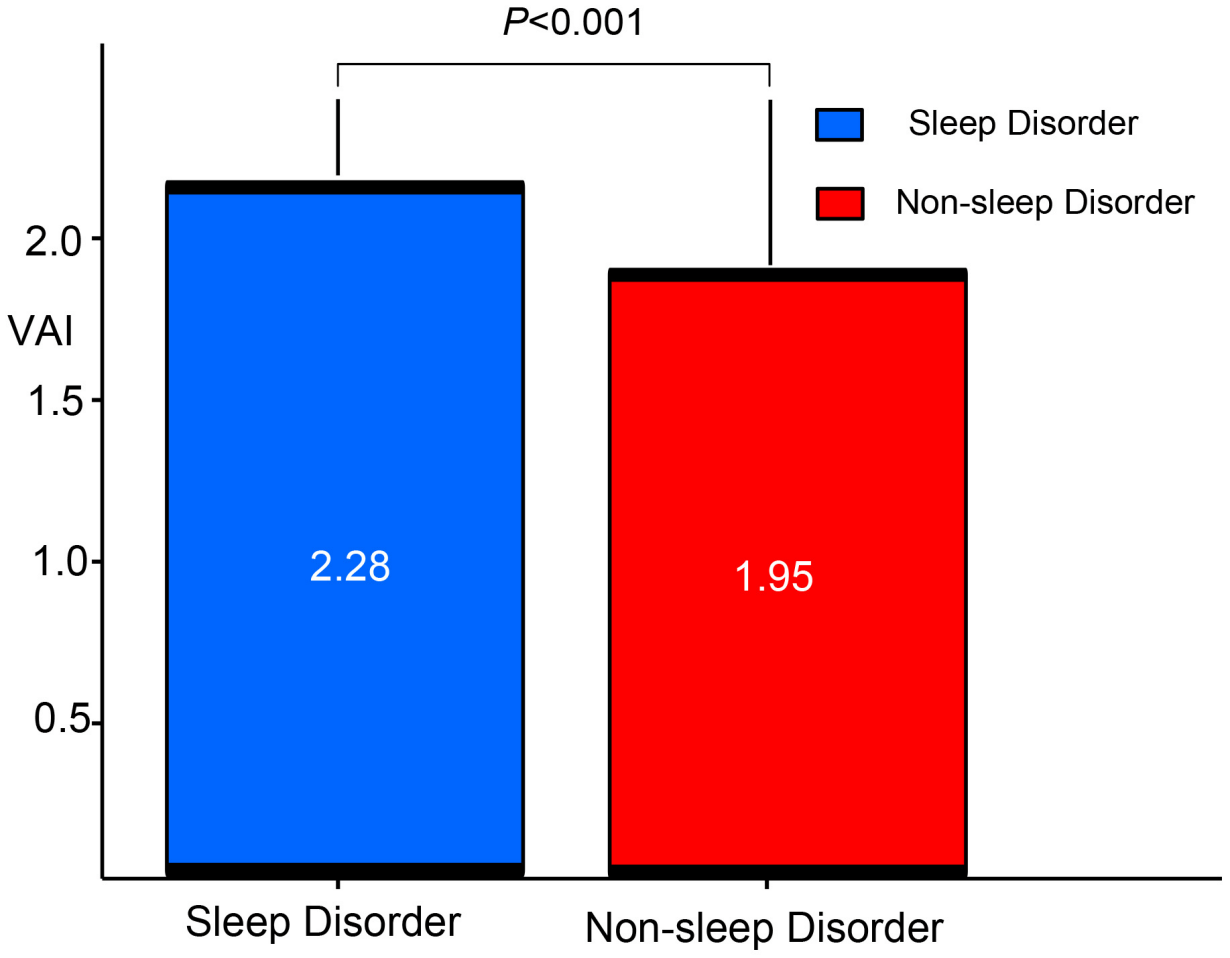
Comparison of VAI between patients with sleep disorders and non-ond rison of Vs. VAI, visceral adiposity index.

Figure 2 highlights the difference in VAI between participants with and without sleep disorders. The results indicate that VAI is significantly higher in those with sleep disorders compared to those without (*p* < 0.001).

Table 2 presents the association between VAI and sleep disorder incidence. Two models were developed using VAI as a continuous variable. In Model 1, which adjusts for age and gender, each unit increase in VAI is linked to a 5% higher likelihood of sleep disorders, with an OR of 1.05 (95% CI: 1.02, 1.07). Model 2, which includes additional adjustments for factors such as race, education, PIR, smoking, alcohol use, caffeine intake, hypertension, diabetes, stroke, and cardiovascular disease, shows an OR of 1.03 (95% CI: 1.00, 1.05). When VAI was categorized into quartiles, participants in the highest quartile (Q4) had a progressively higher prevalence of sleep disorders across all models compared to those in the lowest quartile (Q1) (*P* < 0.021). Overall, these results indicate a positive relationship between VAI and the risk of developing sleep disorders.

**Table 2:** The weighted logistic regression analysis of the association between VAI and sleep disorders.

**Variables**	**Minimally adjusted model (Model 1) OR (95%CI)**	***P*-value**	**Fully adjusted model (Model 2) OR (95%CI)**	***P*-value**
Visceral adiposity index	1.05 (1.02–1.07)	< 0.001	1.03 (1.00–1.05)	0.026
**Visceral adiposity index (Quartile)**				
Q1				
Q2	0.91 (0.79–1.04)	0.2	0.95 (0.81–1.13)	0.6
Q3	1.14 (0.99–1.31)	0.075	1.09 (0.93–1.28)	0.3
Q4	1.33 (1.15–1.53)	< 0.001	1.21(1.03–1.41)	0.021
*P* for trend	< 0.001		< 0.001	

Model 1 adjusted for gender and age.

Model 2 adjusted for gender, age, race, education level, the ratio of family income to poverty, smoking status, alcohol consumption status, hypertension, diabetes, stroke, cardiovascular disease, and dietary caffeine intake. VAI: visceral adiposity index.

### 3.3 Dose-response analysis of VAI on the prevalence of sleep disorders

**Figure 3:**
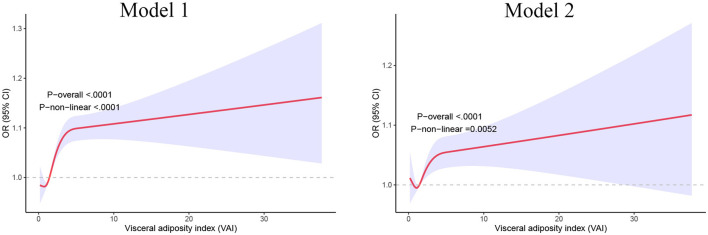
Restricted cubic spline analysis of the relationships between VAI and sleep disorders. Model 1 adjusted for gender and age; Model 2 adjusted for gender, age, race, education level, the ratio of family income to poverty, smoking status, alcohol consumption status, hypertension, diabetes, stroke, cardiovascular disease, and dietary caffeine intake. VAI, visceral adiposity index.

We then applied restricted cubic splines and smooth curve fitting to further examine the relationship between VAI and the prevalence of sleep disorders. As shown in Figure 3, after adjusting for all covariates, we observed a significant positive linear association between higher VAI and increased prevalence of sleep disorders (*p* for linear trend = 0.005).

### 3.4 Subgroup analysis

To further investigate the connection between VAI and sleep disorders in various populations, we performed a stratified analysis by age, gender, and ethnicity. As presented in Table 3, the age-stratified analysis indicated a significant positive association between VAI and sleep disorders in males (OR = 1.01; 95% CI [1.00, 1.02]; *P* = 0.015) and non-Hispanic white participants (OR = 1.01; 95% CI [1.00, 1.01]; *P* = 0.002). Furthermore, males in the highest VAI quartile showed a 6% greater likelihood of having sleep disorders compared to those in the lowest quartile (OR = 1.06; 95% CI [1.02, 1.11]; *P* = 0.01). In the ethnicity-stratified analysis, non-Hispanic white individuals in the highest VAI quartile had a 5% increased chance of experiencing sleep disorders compared to those in the lowest quartile (OR = 1.05; 95% CI [1.01, 1.10]; *P* = 0.028).

**Table 3:** Subgroup analysis of the association between VAI and sleep disorders.

**Exposure**		**Model 1 [OR (95% CI)]**	***p*-value**	**Model 2 [OR (95% CI)]**	***p*-value**
Age subgroup	18–39 years	1.01 (1.00–1.01)	0.027	1.01 (1.00–1.01)	0.13
	18–39 years				
	Q1				
	Q2	0.97 (0.94–1.01)	0.15	0.99 (0.95–1.04)	0.8
	Q3	1.03 (0.98–1.08)	0.2	1.02 (0.98–1.07)	0.3
	Q4	1.04 (0.99–1.10)	0.083	1.04 (0.99–1.09)	0.15
	40–59 years	1.01 (1.00–1.02)	0.005	1.00 (1.00–1.01)	0.094
	40–59 years				
	Q1				
	Q2	0.98 (0.93–1.03)	0.4	1.0 (0.93–1.06)	0.9
	Q3	1.03 (0.98–1.08)	0.3	1.02 (0.97–1.08)	0.4
	Q4	1.09 (1.03–1.14)	0.002	1.05 (0.99–1.11)	0.14
	≥60 years	1.01 (1.00–1.02)	0.13	1.01 (1.0–1.02)	0.3
	≥60 years				
	Q1				
	Q2	1.00 (0.93–1.06)	0.9	0.98 (0.91–1.06)	0.6
	Q3	1.04 (0.97–1.11)	0.3	1.01 (0.94–1.09)	0.8
	Q4	1.05 (0.98–1.13)	0.14	1.02 (0.94–1.10)	0.6
Gender subgroup	Female	1.01 (1.00–1.01)	0.008	1.00 (1.00–1.01)	0.2
	Female				
	Q1				
	Q2	0.97 (0.93–1.01)	0.14	0.96 (0.92–1.00)	0.068
	Q3	1.01 (0.97–1.06)	0.6	0.98 (0.94–1.03)	0.5
	Q4	1.05 (1.01–1.10)	0.022	1.01 (0.96–1.06)	0.8
	Male	1.01 (1.00–1.02)	0.002	1.01 (1.00–1.02)	0.015
	Male				
	Q1				
	Q2	0.99 (0.95–1.03)	0.5	1.02 (0.97–1.06)	0.5
	Q3	1.04 (1.00–1.10)	0.07	1.05 (0.99–1.10)	0.09
	Q4	1.08 (1.03–1.13)	0.003	1.06 (1.02–1.11)	0.01
Race subgroup	Mexican American	1.00 (0.99–1.00)	0.2	1.00 (0.99–1.00)	0.14
	Mexican American				
	Q1				
	Q2	1.00 (0.94–1.06)	>0.9	1.05 (0.98–1.13)	0.2
	Q3	1.00 (0.95–1.05)	>0.9	1.02 (0.95–1.09)	0.6
	Q4	1.00 (0.95–1.06)	>0.9	1.02 (0.96–1.09)	0.5
	Other Hispanic	1.00 (0.99–1.01)	0.6	1.00 (0.99–1.00)	0.2
	Other Hispanic				
	Q1				
	Q2	0.99 (0.91–1.08)	0.8	0.97 (0.89–1.06)	0.5
	Q3	0.97 (0.89–1.05)	0.4	0.95 (0.87–1.03)	0.2
	Q4	1.05 (0.97–1.14)	0.2	0.99 (0.91–1.07)	0.7
	Non-Hispanic White	1.01 (1.01–1.02)	< 0.001	1.01 (1.00–1.01)	0.002
	Non-Hispanic White				
	Q1				
	Q2	0.97 (0.93–1.01)	0.2	0.98 (0.94–1.03)	0.4
	Q3	1.04 (1.00–1.08)	0.072	1.03 (0.99–1.07)	0.2
	Q4	1.08 (1.04–1.13)	< 0.001	1.05 (1.01–1.10)	0.028
	Non-Hispanic Black	1.01 (1.00–1.03)	0.013	1.01 (0.99–1.02)	0.4
	Non-Hispanic Black				
	Q1				
	Q2	1.03 (0.98–1.08)	0.2	1.02 (0.97–1.08)	0.5
	Q3	1.03 (0.99–1.08)	0.2	0.98 (0.94–1.03)	0.4
	Q4	1.10 (1.04–1.16)	0.001	1.07 (1.00–1.14)	0.066
	Other Races	1.00 (0.99–1.02)	0.6	1.00 (0.98–1.02)	>0.9
	Other Races				
	Q1				
	Q2	0.94 (0.86–1.03)	0.2	0.96 (0.88–1.04)	0.3
	Q3	1.02 (0.91–1.14)	0.8	0.99 (0.89–1.10)	0.8
	Q4	0.98 (0.88–1.10)	0.8	0.97 (0.87–1.09)	0.6

Model 1 adjusted for gender and age.

Model 2 adjusted for gender, age, race, education level, the ratio of family income to poverty, smoking status, alcohol consumption status, hypertension, diabetes, stroke, cardiovascular disease, and dietary caffeine intake. VAI, visceral adiposity index.

## 4 Discussion

In this large cross-sectional study, we analyzed 14,021 U.S. participants aged 18 and older to investigate the association between the VAI and sleep disorders. After adjusting for potential confounders, we observed a positive association between VAI and the prevalence of sleep disorders, suggesting that VAI may be a risk factor. As VAI levels increased, so did the risk of sleep disorders. Smoothed curve analysis revealed a linear relationship (*P* for linearity = 0.0052), with the trend becoming steeper as VAI values rose. Subgroup analysis confirmed a strong positive correlation between VAI and sleep disorders in males and non-Hispanic white individuals.

Obesity is increasingly recognized as a major global health issue, with prevalence continually rising. Projections suggest that by 2030, around 2.1 billion people worldwide could be affected by obesity (27, 28). This condition is strongly linked to metabolic disorders such as type 2 diabetes, hypertension, cardiovascular disease, and non-alcoholic fatty liver disease. Although sleep disorders have been extensively studied, their precise mechanisms remain unclear. Obesity is considered a significant risk factor for sleep disturbances, potentially through two primary pathways. First, excess body weight can disrupt normal breathing, leading to obstructive sleep apneaisms remain unclear. Obesity is considered a t nocturnal awakenings and diminished sleep quality. Additionally, fat accumulation around the neck and upper airway may narrow or block the airway during sleep, causing partial or complete breathing interruptions (apnea or hypopnea) (29, 30). Second, abdominal obesity increases intra-abdominal pressure, reduces lung capacity, and is strongly associated with higher visceral fat levels (31). Visceral fat secretes inflammatory and adipose-derived factors, which contribute to systemic inflammation and oxidative stress. Visceral adipose tissue is a major source of pro-inflammatory cytokines like IL-1, IL-6, and TNF-a, which are involved in chronic low-grade inflammation (10). Research suggests that these cytokines play a role in sleep regulation, earning them the label of “sleep regulatory substances (12).” TNF-a and IL-1b secretion follows a circadian rhythm, peaking at night (between 01:00 and 02:00), and is crucial for regulating sleep, particularly slow-wave sleep (SWS) (32, 33). This view is further supported by a study on elderly women in a Spanish community, which found a significant positive correlation between sleep disorders and WC. Since WC is an indicator of visceral fat, this finding supports the hypothesis that visceral fat plays a significant role in the development of sleep disorders (34).

In contrast, sleep disorders may contribute to weight gain and complicate weight loss efforts. Disrupted sleep patterns and frequent awakenings disturb hormone regulation, increasing appetite and cravings for high-calorie foods (35, 36). Several meta-analyses have linked poor sleep quality with obesity, particularly central obesity (37, 38). For instance, a four-year study of 14,000 young people found that shorter sleep duration raised the risk of obesity and increased waist circumference by 1.45 times (39). Additionally, sleep deprivation in children and adolescents is associated with poorer food choices, leading to a higher intake of unhealthy, sugary foods (40). Overall, lack of sleep alters hunger and satiety hormones, affecting food consumption and emotional responses, thus promoting weight gain and obesity-related diseases. The bidirectional link between sleep disorders and obesity creates a vicious cycle, where each condition worsens the other. Shared risk factors, such as unhealthy lifestyle habits, poor diet, and genetic predisposition, further complicate their relationship. In addition, our study's baseline data show that patients without sleep disorders are notably younger, with lower body weight, triglyceride levels, and diabetes prevalence compared to those with sleep disorders. These differences likely arise from several interrelated factors. Aging is often accompanied by metabolic dysregulation, characterized by increased fat accumulation, lipid abnormalities, and insulin resistance, which can heighten the risk of sleep disorders (41, 42). Moreover, elevated body weight and triglyceride levels may contribute to sleep-disordered breathing, such as obstructive sleep apnea, through increased mechanical strain and inflammation (43). The higher diabetes rate in older populations may further reflect a reciprocal relationship between metabolic imbalances and poor sleep quality, with lifestyle factors and the long-term accumulation of metabolic risks also playing important roles (44).

Traditional measures like BMI, while simple and widely used, only capture the weight-to-height ratio and do not reflect fat distribution, especially the distinction between visceral and subcutaneous fat (45). Although waist circumference can indicate abdominal obesity, it does not fully assess visceral fat or its metabolic risks (46). In contrast, the VAI integrates BMI, waist circumference, and lipid parameters (such as triglycerides and HDL cholesterol) to provide a more accurate evaluation of visceral fat accumulation and its associated risks. Studies show that VAI correlates more strongly with cardiovascular and metabolic abnormalities than BMI or waist circumference alone, suggesting it is a more sensitive marker (47, 48). Moreover, while devices like InBody offer detailed body composition analysis, their high cost and strict standardization make them less suitable for large-scale studies. As a computed index, VAI is cost-effective, easy to implement, and serves as a non-invasive alternative to CT or MRI (49). Since its introduction in 2010, VAI has been validated for predicting diabetes, cardiovascular disease, and other health risks (23, 50).

In this study, we compared our results with existing literature and found that VAI outperforms individual lipid components in predicting cardiovascular diseases, with a clear link to sleep disorders. Our analysis demonstrated a positive linear relationship between VAI and sleep disorders. Prior research has identified VAI as a useful marker for predicting insulin resistance in obstructive sleep apnea patients (51, 52). Our subgroup analysis revealed that elevated VAI levels are linked to a higher risk of sleep disorders in men, but not in women. This gender-specific difference might stem from variations in metabolism, endocrine function, and hormone levels (53, 54). These factors likely contribute to the observed differences in the diagnosis and presentation of sleep disorders between men and women.

This study has several important strengths. First, it is the first to explore the association between VAI and the risk of sleep disorders, making a valuable contribution to the field. Second, the NHANES data, which follows strict quality control protocols, ensures reliable results. Third, VAI allows for a detailed assessment of visceral fat distribution without the high costs, radiation exposure, and complexity linked to CT and MRI scans, making it more practical for clinical and screening purposes. Fourth, the large sample size offers sufficient statistical power for subgroup analysis.

Despite the strengths of this study, several limitations must be acknowledged. First, self-reported data on cardiovascular disease, stroke, and sleep disorders may be subject to recall or reporting bias since these conditions were not independently verified. Second, our analysis relies on the NHANES database, where sleep disorders were determined by a general inquiry per the NHANES protocol, rather than using validated, disorder-specific questionnaires (e.g., MCTQ or MEQ). Consequently, we could only ascertain whether a participant had ever been diagnosed with a sleep disorder, without distinguishing among its subtypes, which limits the clinical interpretation of our results. Lastly, the cross-sectional design, with exposure and outcome measured simultaneously, impedes the establishment of temporal relationships and limits causal inferences. Future research should include large-scale prospective cohort studies and randomized controlled trials employing validated, disorder-specific assessment tools to further confirm these findings.

## 5 Conclusion

In summary, this study using NHANES data reveals a strong link between higher VAI levels and increased risk of sleep disorders, indicating that VAI could be a useful predictor. Managing VAI levels may help slow the progression of sleep disorders and serve as a preventive measure. For those with elevated VAI, adjustments in diet and exercise are recommended to lower the risk of sleep disorders. These results highlight the need to integrate VAI assessment into clinical practice to guide personalized interventions and enhance outcomes for individuals at high risk.

## Data Availability

The original contributions presented in the study are included in the article/Supplementary material, further inquiries can be directed to the corresponding author/s.
